# IL-33-mediated mast cell and eosinophil function requires isoprenylation

**DOI:** 10.3389/fimmu.2025.1662170

**Published:** 2025-12-11

**Authors:** Jason R. Burchett, Aditya Kotha, Destiny T. Davis, Kaitlyn G. Jackson, Jordan M. Dailey, Tania D. Maldonado, Tamara T. Haque, Zakaria Y. Hussain, John M. Ching, Pamela Frischmeyer-Guerrerio, Said M. Sebti, John J. Ryan

**Affiliations:** 1 Department of Microbiology and Immunology, Virginia Commonwealth University, Richmond, VA, United States; 2 School of Life Sciences and Sustainability, Virginia Commonwealth University, Richmond, VA, United States; 3 Department of Pharmaceutical Sciences, College of Pharmacy, University of Michigan, Ann Arbor, MI, United States; 4 Wells Center for Pediatric Research, Department of Pediatrics, Indiana University, Indianapolis, IN, United States; 5 National Institute of Allergy and Infectious Diseases (NIAID), National Institutes of Health, Bethesda, MD, United States; 6 Department of Pharmacology and Toxicology, Virginia Commonwealth University, Richmond, VA, United States

**Keywords:** IL-33, inflammation, simvastatin, mast cells, eosinophils, isoprenylation

## Abstract

**Introduction:**

Allergic disease is a common and symptomatically heterogeneous group of inflammatory disorders marked by overactive Th2 and mast cell (MC) responses along with eosinophil infiltration. Treatment options require continual assessment due to breakthrough symptoms on standard regimens. One approach to improved therapy is drug repurposing. Our lab previously showed that cholesterol-lowering statin drugs can suppress IgE-mediated mast cell function by inhibiting protein isoprenylation, a pathway using cholesterol biosynthesis intermediates. Additionally, mast cells are activated by the alarmin IL-33, released by epithelial cells after contact with cellular stressors. We hypothesized that IL-33-mediated mast cell function can be inhibited by disrupting isoprenylation via statins or the dual farnesyltransferase (FT) geranylgeranyltransferase-1 inhibitor, FGTI-2734.

**Methods:**

We used IL-33 to stimulate mast cells and eosinophils *in vitro* and inhibited their function using simvastatin and FGTI-2734. Using primary mast cells and eosinophils, we measured cytokine production by ELISA and qPCR. Flow cytometry and western blots were used to measure phosphorylation of IL-33 signaling components, and eosinophil migration. Human mast cells were assessed by ELISA for cytokine inhibition. Lastly, a murine model of IL-33 induced peritonitis was used to assess the effects of isoprenylation inhibition on eosinophil and neutrophil influx.

**Results:**

We show simvastatin and FGTI-2734 suppressed IL-33-mediated cytokine protein and mRNA production in primary murine mast cells from the C57BL/6 strain. Simvastatin effects were lost on mast cells from the 129/SvJ strain and were inconsistent among primary human mast cells. In contrast, FGTI-2734 inhibited IL-33-induced cytokine production by mast cells on the 129/SvJ strain and among human donors. Simvastatin and FGTI-2734 also inhibited IL-33-induced cytokine production and chemokine-induced migration of C57BL/6 primary eosinophils. Simvastatin and FGTI-2734 had no effect on expression of the IL-33 receptor, ST2, suggesting that inhibition occurs at a step in IL-33 signaling. Importantly, FGTI-2734 significantly reduced eosinophil and neutrophil influx in a model of IL-33-induced peritonitis, whereas simvastatin had no effect.

**Discussion:**

These findings indicate that targeting FT and GGT-1 is a viable target in IL-33-induced inflammation.

## Introduction

Allergic diseases are a common and varied set of disorders affecting more than one-third of Americans. For example, the CDC reported that in 2021, nearly 40% of the US population reported seasonal allergies, eczema, or food allergies (1). Additionally, nearly 25M Americans report having asthma, most of which is allergic (2). Numerous treatment options are available, including topical, inhaled, or systemic corticosteroids, short- and long-acting β-agonists, and newer targeted biologics (3). Despite continuous reassessment and treatment options, symptoms persist or recur for many. For example, 56% of asthma patients suffer severe asthma exacerbations when using both control and rescue medications (4, 5). Even with the newest biologics like dupilumab, severe eczema can be challenging to manage due to exacerbations and adverse drug events (6). Therefore, alternative or supplemental treatment options are needed for allergic disease.

Statins are a widely prescribed class of lipid-lowering medications used to manage hypercholesterolemia and prevent cardiovascular disease (7). Statins inhibit the rate-limiting enzyme 3-hydroxy-3-methylglutaryl-coenzyme A reductase (HMGCR), effectively reducing cholesterol biosynthesis in the liver, thereby lowering low-density lipoprotein (LDL) cholesterol levels (8). In addition to their lipid-lowering capabilities, statins have pleiotropic effects, including anti-inflammatory properties (Reviewed in (9)). Importantly, statins exhibit protective effects against asthma. Across four large-scale studies of statin users with asthma, statins were shown to reduce severe exacerbations, corticosteroid use, and asthma-related emergency department visits and hospitalizations (10–13). It has been hypothesized that these anti-inflammatory effects are partly mediated by suppressing protein isoprenylation rather than lowering cholesterol (reviewed in (14)).

**Figure 1:**
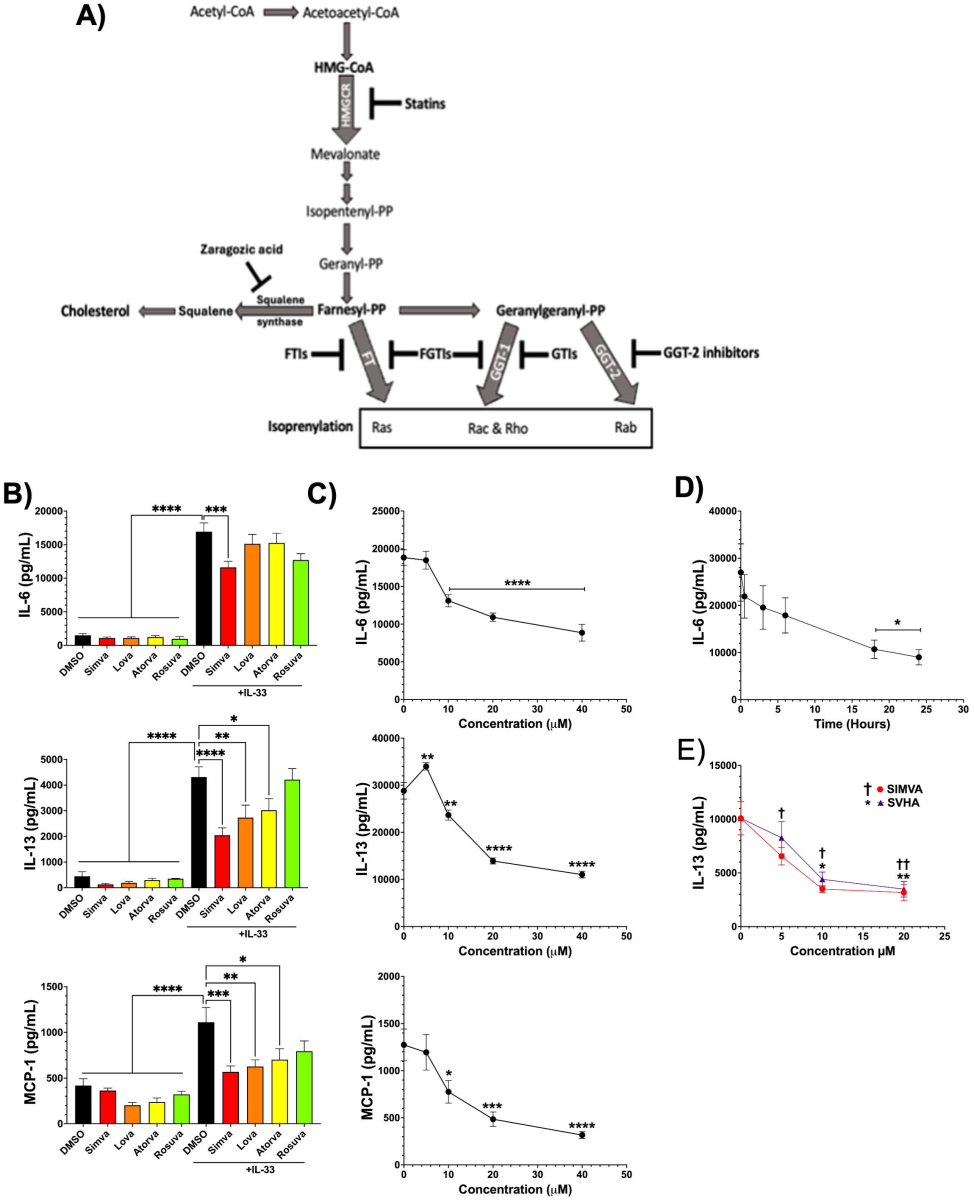
Simvastatin inhibits IL-33-mediated mast cell function. **(A)** Mevalonate pathway schematic. **(B)** C57BL/6J BMMC were treated with atorvastatin (20μM), lovastatin (20μM), rosuvastatin (20μM), or simvastatin (20μm) for 24 hours. **(C)** C57BL/6J BMMC were treated with simvastatin at 0, 5, 10, 20, and 40μM for 24 hours. **(D)** C57BL/6J BMMC were treated with simvastatin (20μM) at 0, 0.5, 3, 6, 18, and 24 hours. **(E)** C57BL/6J BMMC were treated with simvastatin or simvastatin hydroxy acid (SVHA) at 0, 5, 10, and 20μM for 24 hours. In all experiments, cells were stimulated with IL-33 (100 ng/mL) for 16 hours and supernatant cytokines were measured by ELISA. Icons represent the mean standard error of each sample set. Data shown in part (B) are from 2 (rosuvastatin) or 3 experiments. Parts **(C, D)** are representative of at least 4 independent experiments. Part **(E)** is from 3 independent BMMC populations analyzed in replicates by 2-way ANOVA with Tukey’s post-hoc test and multiple comparisons correction. In (C–E), all comparisons are relative to the “0” control group. *p< 0.05, **p< 0.01, ***p< 0.001, ****p<0.0001, and no markers meaning non-significant. †p< 0.05, ††p<0.01 († indicated SIMVA comparison to “0” control group).

Isoprenylation is the post-translational modification of proteins that covalently attaches 15-carbon farnesyl and 20-carbon geranylgeranyl lipid groups to the protein C-terminus, via farnesyl and geranylgeranyl transferases, respectively (**Figure 1A**) (15). These lipids are attached to the cysteine thiol group in a C-terminal CaaX motif, where ‘C’ represents cysteine, ‘a’ is an aliphatic amino acid, and ‘X’ determines the type of isoprenoid added. Isoprenylation facilitates anchoring proteins to cell membranes, mediating their localization, interactions, and functions (16, 17). This modification is crucial for the activity of various proteins, such as the Ras and Rho family of small GTPases, which play essential roles in signal transduction, cell growth, and cytoskeletal organization. Consequently, dysregulation of isoprenylation is implicated in numerous diseases, such as cancer and cardiovascular disorders, making it a significant area of study for therapeutic intervention (18).

Interleukin-33 (IL-33) is a pleiotropic cytokine of the IL-1 family that plays a crucial role in immune regulation and inflammation (19). Unlike most cytokines, IL-33 is a chromatin-associated nuclear protein that is abundant in structural cells like epithelial and endothelial cells (20). Upon cell damage, IL-33 is released and acts as a danger signal, or alarmin, that alerts immune cells to damage potentially caused by infection (19). Thus IL-33 is categorized as both a cytokine and a damage-associated molecular pattern (DAMP). By binding to its surface receptor ST2, IL-33/ST2 signals through a MyD88-dependent pathway activating MAP kinases and Akt, leading to NFκB-mediated transcription of inflammatory cytokines (21). Originally described as an activator of Th2 cells and mast cells, IL-33 was subsequently shown to potently stimulate type 2 innate lymphoid cells and eosinophils, making it a central player in allergic inflammation (22, 23).

Because isoprenylated Ras family proteins promote activation of MAP kinases and Akt in many cell signaling cascades, it is logical that suppressing isoprenylation could reduce IL-33 signaling. In this study, we show that simvastatin inhibits IL-33-mediated cytokine release and transcription from primary cultures of mast cells and eosinophils. Chemokine-induced eosinophil migration was also suppressed. These effects were phenocopied by FGTI-2734-mediated inhibition of isoprenylation (24). Because suppressing cholesterol synthesis downstream of the isoprenylation pathway had no effect on mast cell cytokine release, we conclude that simvastatin acts by reducing isoprenylation rather than by lowering cholesterol. Importantly, we previously found that statin effects on mast cells vary greatly with mouse strain, likely due to drug-induced HMGCR upregulation (25, 26). Consistent with this, mast cells from the 129/SvJ mouse strain were completely resistant to simvastatin, while FGTI-2734 inhibition remained intact. Interestingly, an IL-33-induced peritonitis model that elicits eosinophil and neutrophil influx showed that FGTI-2734, but not simvastatin, could greatly reduce inflammation. Similarly, we found that FGTI-2734 but not simvastatin consistently suppressed IL-33 effects on mast cells from human donors.

Collectively, these findings support targeting isoprenylation as a therapeutic intervention in allergic disease. Our data also suggest that the positive impact of statins on allergic disease noted in the literature may be related to effects on isoprenylation rather than reduced cholesterol.

## Materials and methods

### Animals

C57BL/6J mouse breeding pairs were purchased from The Jackson Laboratory. Breeding colonies were maintained in a specific pathogen-free facility. Mouse strain 129/SvJ was purchased from The Jackson Laboratory and used upon arrival. Experiments used age- and sex-matched mice housed in the same facility. Bone marrow was extracted from mice at a minimum of 8 weeks of age. *In vivo* models were conducted with mice 10 weeks or older under a protocol approved by the Virginia Commonwealth University Institutional Animal Care and Use Committee.

### Mouse cells

Mouse bone marrow-derived mast cells (BMMC) were grown by culturing bone marrow extracted from femurs in complete RPMI (cRPMI) 1640 medium (InVitrogen Life Technologies) containing the following supplements purchased from Corning: 10% fetal bovine serum (FBS), 2mM L-glutamine, 100U/ml penicillin, 100µg/mL streptomycin, 1mM sodium pyruvate, and 1mM HEPES. Cultures were supplemented with IL-3 and SCF at final concentrations of 10 ng/ml each. BMMC were used on culture day 21 or later and consisted of ~90% mast cells based on cell surface staining for c-Kit, FcϵRI and ST2 (**Supplementary Figure 1**).

Peritoneal mast cells (PMC) were obtained from peritoneal cavity lavage using PBS containing 1mM EDTA. Cells were cultured in cRPMI supplemented with IL-3 and SCF at concentrations of 10 ng/mL and 20 ng/mL, respectively, for at least 10 days and consisted of ~90% mast cells based on cell surface staining for c-Kit and FcϵRI.

Mouse bone marrow-derived eosinophils (BMDE) were grown by culturing bone marrow extracted from femurs in cRPMI 1640 medium containing the following supplements: 20% FBS, 25mM HEPES, 100U/mL penicillin, 100µg/mL streptomycin, 1x non-essential amino acids, 1mM sodium pyruvate, 1mM GlutaMAX, and 55µM 2-mercaptoethanol (2-ME). Cultures were supplemented with FMS-related tyrosine kinase 3 ligand (FLT3L) (100 ng/mL) and SCF (100 ng/mL) for 4 days, then FLT3L and SCF were replaced with recombinant IL-5 (10 ng/mL). Cultures were used on days 10 to 16. Viability was measured by Trypan blue staining prior to use and was >90%.

### Human mast cell cultures

Participants were recruited onto clinical protocol 15-I-0162 (NCT 02504853) approved by the Institutional Review Board of the NIH. Informed consent was obtained. Peripheral blood mononuclear cells were isolated from peripheral blood from healthy volunteers by Percoll gradient centrifugation. CD34+ cells were enriched using EasySep negative selection Human Progenitor Cell Enrichment Kit (StemCell) using manufacturers’ instruction. Progenitor cells were plated at 0.5x10^6^ cells/ml and cultured in StemPro™-34 serum-free media (Thermofisher) with 30 ng/mL of IL-3 (Peprotech) for 2 weeks and 100 ng/mL of both IL-6 (Peprotech) and SCF (Peprotech) for the entire duration of the culture. Six weeks after culture, cells were analyzed for c-KIT and FcϵRI expression by flow cytometry and then used when >95% of the population was double-positive for these markers. For IL-33 stimulation, cells were washed and replated in StemPro™-34 serum-free media with 100 ng/mL SCF at 1x10^6^ cells/ml with vehicle control or inhibitors at the indicated dose for 24 hours, then cells were stimulated with 100 ng/mL of recombinant IL-33 (Biolegend) for 16 hours overnight. Supernatant was collected to measure IL-13 by ELISA (Biolegend).

### Cytokines and reagents

Recombinant mouse IL-3, SCF, and IL-33 and antibodies against ST2 (clone DIH9, catalog# 145306), c-KIT (clone 2B8, catalog# 105827), and phospho-p38 (clone A16016A, catalog# 690203) were purchased from Biolegend. Antibodies against ERK (clone L34F12, catalog# 4696), phosphor-ERK (catalog# 91015), JNK (clone 56G8, catalog# 9255), phospho-JNK (clone G8, catalog# 9255S), p65 (clone L8F6, catalog# 6956), and phospho-p65 (clone 93H1, catalog# 3033) were purchased from Cell Signaling Technology (Danvers, MA). Simvastatin, simvastatin hydroxy acid, lovastatin, atorvastatin, rosuvastatin, zaragozic acid, and FGTI-2734 were purchased from Cayman Chemical. Mevalonic acid was purchased from Millipore-Sigma. Farnesyl transferase inhibitor (FTI-2153) and geranylgeranyl transferase inhibitor (GGTI-2417) were synthesized as described previously [150,151]. Simvastatin hydroxy acid was resuspended in cRPMI. Simvastatin, lovastatin, atorvastatin, rosuvastatin, FGTI-2734, FTI, and GGTI were resuspended in dimethyl sulfoxide (DMSO). When used *in vivo*, FGTI-2734 was resuspended in 2-Hydroxypropyl-β-cyclodextrin (2HBC) from Milipore-Sigma.

### Secreted cytokine measurements

BMMCs, PMCs, and BMDEs were resuspended at 1x10^6^ cells/mL in their respective medium with appropriate growth factors as described above. Cells were treated with the indicated inhibitors for 24 hours unless otherwise indicated and stimulated with IL-33 (100 ng/mL) for 16 hours, unless otherwise indicated. Cell supernatants were collected and assessed for IL-6, TNFα, and MCP-1 content using ELISA kits from Biolegend (San Diego, CA) or IL-13 and MIP-1α using ELISA kits from Peprotech. ELISAs were developed using BD OptEIA regents from BD Biosciences and read on a Multiskan™ FC Microplate Photometer (Thermo Scientific).

### Quantitative RT-PCR

BMMCs or BMDEs were stimulated by IL-33 (100ng/mL) for 5 hours and RNA was harvested using TRIzol reagent (Life Technologies) per the manufacturer’s protocol. Purity of the extracted nucleic acid was measured using a Nanodrop 1000 UV-Vis Spectrophotometer (Thermo Scientific). Synthesis of cDNA was done using qScript cDNA Synthesis Kit (Quanta Bioscience) per the manufacturer’s protocol. To determine IL-6, IL-13, TNFα, and GAPDH mRNA expression, real time quantitative PCR (RT-qPCR) was performed with the CFX96 Touch Real-Time PCR Detection System (Bio-Rad) and PerfeCTa SYBR Green SuperMix (Quanta Biosciences). The following amplification conditions were used: a heat activation step of 95°C for 2 minutes, followed by 40 cycles of 95°C for 15 seconds, 55°C for 30 seconds, 60°C for 1 minute, A melting curve analysis was performed between 55°C and 95°C. Primers used included: IL-6 (forward: 5′-TCCAGTTGCCTTCTTGGGAC-3′, reverse: 5’-TCCAGTTGCCTTCTTGGGAC-3′), IL-13 (forward: 5’-ATGGCGTCTGGGTGACTGCAGTCC-3’, Reverse: 5’-GAAGGGGCGTGGCGAAACAGTTGC-3’), TNFα (forward: 5’-AGCACAGAAAGCATGATCCGC-3’ reverse: 5’-TGCCACAAGCAGGAATGAGAAG -3’), and GAPDH (forward: 5’-GATGACATCAAGAAGGTGGTG-3’, reverse: 5’-GCTGTAGCCAAATTCGTTGTC-3’).

### Receptor expression

BMMCs were resuspended at 1x10^6^ cells/mL in cRPMI with IL-3 and SCF (10 ng/mL) and were treated with the indicated inhibitors for 24 hours. Cells were stained for c-Kit and ST2. Mean fluorescence intensity was measured by flow cytometry. Gating strategy was as follows: cells were gated with doublet exclusion via FSC-H and FSC-A, then gated on FSC-H versus SSC-H, then isotype IgG controls were used to set gates for c-Kit and ST2 positive cells.

### Phosphorylation measured by flow cytometry

BMMCs were resuspended at 1x10^6^ cells/mL in cRPMI with IL-3 and SCF (10 ng/mL) and were treated with the indicated inhibitors for 24 hours. Cells were stimulated with IL-33 (100 ng/mL) for indicated times (0, 1, 5, 15, or 30 minutes) then collected and fixed using 4% formaldehyde (Macron Fine Chemicals) for 20 minutes at room temperature. Cells were permeabilized with 1X PermBuffer (ThermoFisher) and stained for phosphorylated (p-) p38 overnight at 4 °C. Mean fluorescence intensity was measured by flow cytometry. Gating strategy was as follows: cells were gated by single cell exclusion using FSC-H and FSC-A and then isotype IgG controls were used to set gates for p-p38 positive cells.

### Western blot

Cells were resuspended in medium and placed in a 37°C heat block and stimulated with IL-33 (100 ng/mL) for times indicated in the experiments. Phosphorylation was then halted by adding ice-cold PBS in the samples. Cells were pelleted and washed with ice-cold PBS and resuspended in RIPA lysis buffer [10 mM tris-HCl (pH 7.5), 300 mM NaCl, 1mM EDTA, 1% Triton X-100, 1% SDS, and 0.1% sodium deoxycho-late] containing protease inhibitor cocktail (Millipore Sigma, catalog no. P8340, 1:100 dilution). Samples incubated on ice for 30 minutes. Lysates were centrifuged at 20,000g for 30 min at 4 °C, supernatants were collected, and a Pierce BCA Protein Assay (ThermoFisher Scientific, catalog no. 23225) was performed. Western blot analysis was performed as described previously (27). In brief: 40μg protein per lane was loaded and a 4-20% SDS-polyacrylamide gel (BioRad) was used for gel electrophoresis. Proteins were then transferred for 45 minutes at 25 V onto nitrocellulose paper. Blots were blocked for 30 minutes with 50% Casein buffer in TBS. Membranes were then incubated with appropriate antibody concentrations as recommended by the manufacturer overnight in 4 °C. Membranes were washed and then incubated with appropriate secondary antibody for 1hr.Blots were visualized and quantified using a LiCor Odyssey CLx infrared imaging system (Lincoln, NE).

### Migration assay

BMDE were resuspended at 1x10^6^ cells/mL in eosinophil culture medium described earlier, supplemented with IL-5 (10ng/mL). Cells were treated with indicated inhibitors for 24 hours and treatment was reapplied with any medium change. For migration, 24-well Transwell™ culture plates with 5μm pore size were used (Corning, Cat# 3421). No additional coating was added to the membranes. In the bottom chamber, 700μL of RPMI + 5% BSA was added. In the top chamber 0.5x10^6^ BMDE in 200μL of RPMI + 5% BSA was added. Cells were starved of IL-5 for 2 hours prior to addition of chemokines. After starvation, CCL24 (Peprotech, Cat# 250-22) (14.6 nM) was added to the bottom chamber. Cells were incubated for 2.5 hours at 37°C in a humidified incubator with 5% CO_2_. Bottom chamber supernatants were collected, and cells were counted using a flow cytometer, acquiring events for 30 seconds at a time resolution of 0.1 seconds.

### Peritonitis model

Age-matched C57BL/6J mice (10–16 weeks old) received IP injections of 200µL sterile PBS with vehicle, FGTI-2734 (10 mg/kg), or simvastatin (10 mg/kg) for 5 days. Starting on day 2, IL-33 (0.5µg) was added to the daily injections. Four hours after the final injection of IL-33, mice were euthanized for collection of peritoneal lavages. Peritoneal lavage cells were counted on a Luna II Automatic Cell Counter and assessed by flow cytometry using a BD FACSCelesta. Gating strategy was as follows: cells were gated by single cell exclusion using FSC-H and FSC-A, granulocytes were gated by FSC-A and SSC-A. CD45^+^ (clone 30-F11, Biolegend 103112) cells were then gated on specific cell types CD11b^+^ (clone M1/70, Biolegend 101206), SiglecF^+^ (clone S17007L, Biolegend 1555099) (eosinophils), or CD11b^+^, Ly6G^+^ (clone AL21, Biolegend 553104) (neutrophils). FGTI-2734 was resuspended in 2-hydroxypropyl-b-cyclodextrin, simvastatin was resuspended in DMSO.

### Statistical analysis

GraphPad Prism software v10 was used to calculate p values. Data in each figure are expressed as mean ± standard error of mean (SEM) with statistical significance: *p< 0.05, **p< 0.01, ***p< 0.001, ****p<0.0001, and n.s. or absent markers meaning non-significant. Biological replicates for *in vitro* experiments were cell populations cultured from separate mice. Biological replicates for *in vivo* experiments were individual mice. Technical replicates were conducted for all experiments and consisted of duplicates or triplicates of each sample. Unless otherwise stated, comparisons of 2 groups were done by Student’s t-Test; for 3 or more groups, ANOVA was used with Dunnett’s *post-hoc* test and corrections for multiple comparisons.

## Results

### IL-33-mediated mast cell function is inhibited by simvastatin

We previously showed that statins inhibit IgE-mediated MC function, and that this inhibition is due to disruption of the isoprenylation pathway (25, 26). IL-33 is a potent pleiotropic cytokine that stimulates mast cells through the surface receptor ST2 (21). Considering statins’ ability to inhibit IgE-mediated mast cell function, we hypothesized that statins would also suppress IL-33-mediated mast cell function.

We tested different statins, including the first-generation lovastatin and its derivative simvastatin and the second-generation atorvastatin and rosuvastatin (28–30). To determine their ability to inhibit IL-33-mediated MC responses, BMMC were pretreated with statins for 24 hours and stimulated with IL-33 (100ng/mL) for 16 hours. Simvastatin consistently suppressed IL-6, IL-13, and MCP-1 supernatant cytokines measured by ELISA (**Figure 1B**). Lovastatin and atorvastatin only suppressed IL-13 and MCP-1, while rosuvastatin showed no significant inhibition of any cytokine (**Figure 1B**). Simvastatin exhibited the greatest magnitude of inhibition among all cytokines measured; therefore, further studies focused on this drug. Simvastatin was most effective at inhibiting cytokines after 24-hour pretreatment, and inhibition increased with the greater drug concentrations (**Figures 1C, D**).

Simvastatin is a prodrug that requires metabolism to its active form simvastatin hydroxy acid, typically by CYP3A4 (31),. Therefore, we did a direct comparison of simvastatin and simvastatin hydroxy acid to determine if the active metabolite was more effective. Simvastatin and simvastatin hydroxy acid inhibited IL-13 production equally in C57BL/6J BMMC (**Figure 1E**), suggesting BMMCs effectively metabolize simvastatin.

### Simvastatin acts on HMGCR, reducing access to isoprenyl lipids

**Figure 2:**
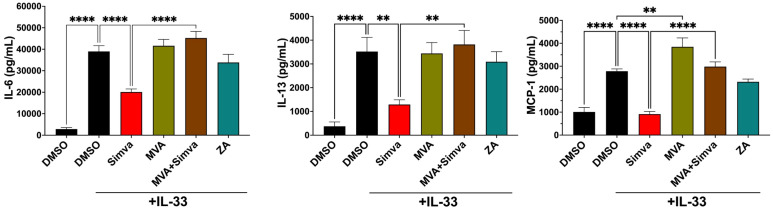
Simvastatin effects are due to inhibition of isoprenylation and not by targeting cholesterol synthesis. C57BL/6J BMMC were treated with simvastatin (20μM), mevalonic acid (MVA) (1mM), or zaragozic acid (ZA) (20μM) as indicated for 24 hours, then stimulated with IL-33 (100ng/mL) for 16 hours. Supernatant cytokines were determined by ELISA. Bars represent the mean standard error of each sample set. Data shown are from 3 experiments, each using 3 independent BMMC populations analyzed in replicates by 1-way ANOVA with Dunnett’s post-hoc test and multiple comparisons correction. **p< 0.01, ****p< 0.0001, and no markers meaning non-significant.

The variable effects of different statin drugs could suggest that simvastatin is acting off-target. To test this, we determined if simvastatin-mediated suppression could be reversed by restoring the HMGCR product, mevalonate. We observed that adding mevalonic acid (MVA) to cultures had no effect on IL-6 and IL-13 release but did elevate MCP-1. More importantly, MVA addition rescued cytokine production when given simultaneously with simvastatin, indicating simvastatin was likely acting on HMGCR (**Figure 2**).

Downstream of HMGCR, cholesterol synthesis branches at farnesyl pyrophosphate, which can be converted to geranylgeranyl pyrophosphate in the isoprenyl lipid pathway or metabolized by squalene synthase in the cholesterol pathway (**Figure 1A**). We found that inhibiting squalene synthase with zaragozic acid (ZA) prior to IL-33 challenge had no effect on IL-6, IL-13, or MCP-1 secretion (**Figure 2**). This suggested that inhibiting cholesterol synthesis is not the major mechanism by which simvastatin reduces cytokine production. None of the interventions tested impacted cell viability (**Supplementary Figure 2**). Overall, these findings indicate that simvastatin acts on its intended target and reduces production of isoprenyl lipids needed for FT and GGT-1 function.

### Isoprenylation inhibition mimics the effects of simvastatin

**Figure 3:**
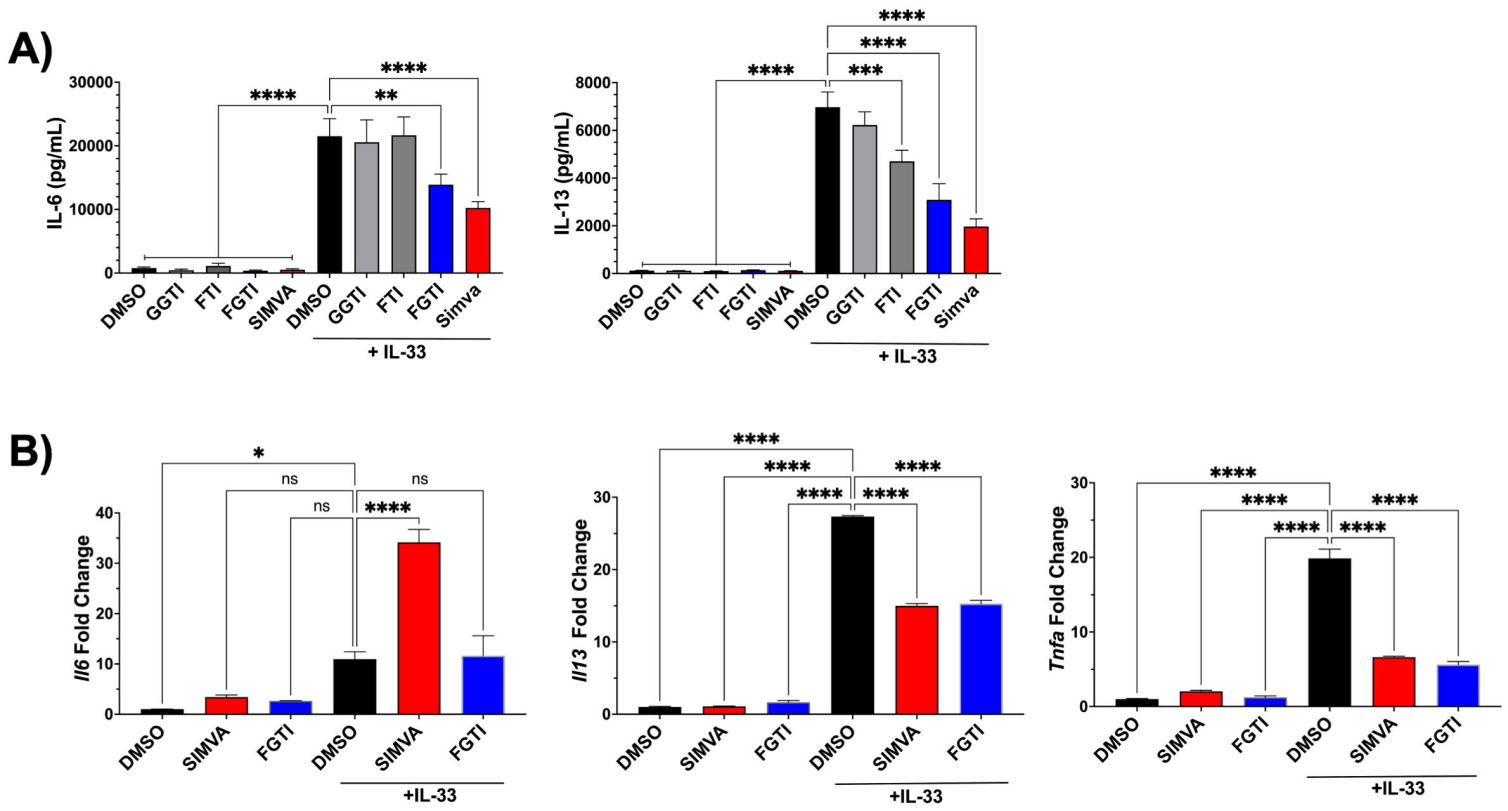
Inhibiting both isoprenylation transferases consistently reduces IL-33-mediated cytokine production. **(A)** C57BL/6J BMMC were treated with GGTI-2417 (10μM), FTI-2153 (10μM), FGTI-2734 (10μM), or simvastatin (20μM) for 24 hours, then stimulated with IL-33 (100ng/mL) for 16 hours. Supernatant cytokines were determined by ELISA. **(B)** C57BL/6J BMMC were treated with simvastatin (20μM) or FGTI-2734 (10μM) for 24 hours and stimulated with IL-33 (100ng/mL) for 5 hours. Transcription was measured by RT-qPCR. Bars represent the mean standard error of each sample set. Data shown in **(A)** are from 3 independent experiments using independent BMMC populations analyzed in replicates. Data in **(B)** are from 3 independent BMMC populations analyzed in replicates by 1-way ANOVA with Dunnett’s post-hoc test and multiple comparisons correction. *p< 0.05, ***p< 0.001, ****p< 0.0001, and no markers meaning non-significant.

Isoprenylation is the post-translational modification of proteins that includes the addition of 15-carbon farnesyl isoprenoids by farnesyltransferase (FT) and 20-carbon geranylgeranyl isoprenoids by geranylgeranyl transferase (GGT) -1 and -2 (15). To test the importance of isoprenylation in IL-33 mediated MC function, we used antagonists of FT, GGT-1, and the dual FT/GGT-1 inhibitor, FGTI-2734 (16, 24). We also directly compared these antagonists to simvastatin. All inhibitors were given for 24 hours prior to IL-33 stimulation and cytokines were measured after 16 hours of activation. FTI failed to inhibit IL-6 release but did reduce release of IL-13. GGTI failed to inhibit IL-6 or IL-13 secretion. FGTI-2734 and simvastatin inhibited both IL-6 and IL-13 release (**Figure 3A**). We also tested increasing concentrations of FGTI-2734 and found that 10μM was effective (**Supplementary Figure 3**). These data suggest that inhibiting isoprenylation can mimic statin effects and that optimal inhibition requires both FT and GGT-1 suppression. Notably, simvastatin and FGTI-2734 also inhibited IL-33-mediated cytokine production in peritoneal mast cells, indicating that our findings are consistent in mast cells that differentiate *in vivo* (**Supplementary Figure 4**).

Since simvastatin and FGTI-2734 efficiently suppressed cytokines at the protein level, we determined if cytokine transcription was impacted by these drugs. We measured *Il6*, *Il13*, and *Tnfa* expression by RT-qPCR after a 24-hour pretreatment with simvastatin or FGTI-2734, followed by stimulation with IL-33 for 5 hours. Both drugs significantly suppressed *Il13* and *Tnfa* (**Figure 3B**). Interestingly, even though IL-6 protein secretion was consistently suppressed, *Il6* mRNA expression was significantly increased by simvastatin and unchanged by FGTI-2734 (**Figure 3B**). This suggests that IL-33-induced cytokine production was inhibited partly by reducing mRNA, with a separate mechanism reducing IL-6 secretion.

### The effects of simvastatin may be more dependent on genetic background than FGTI-2734

**Figure 4:**
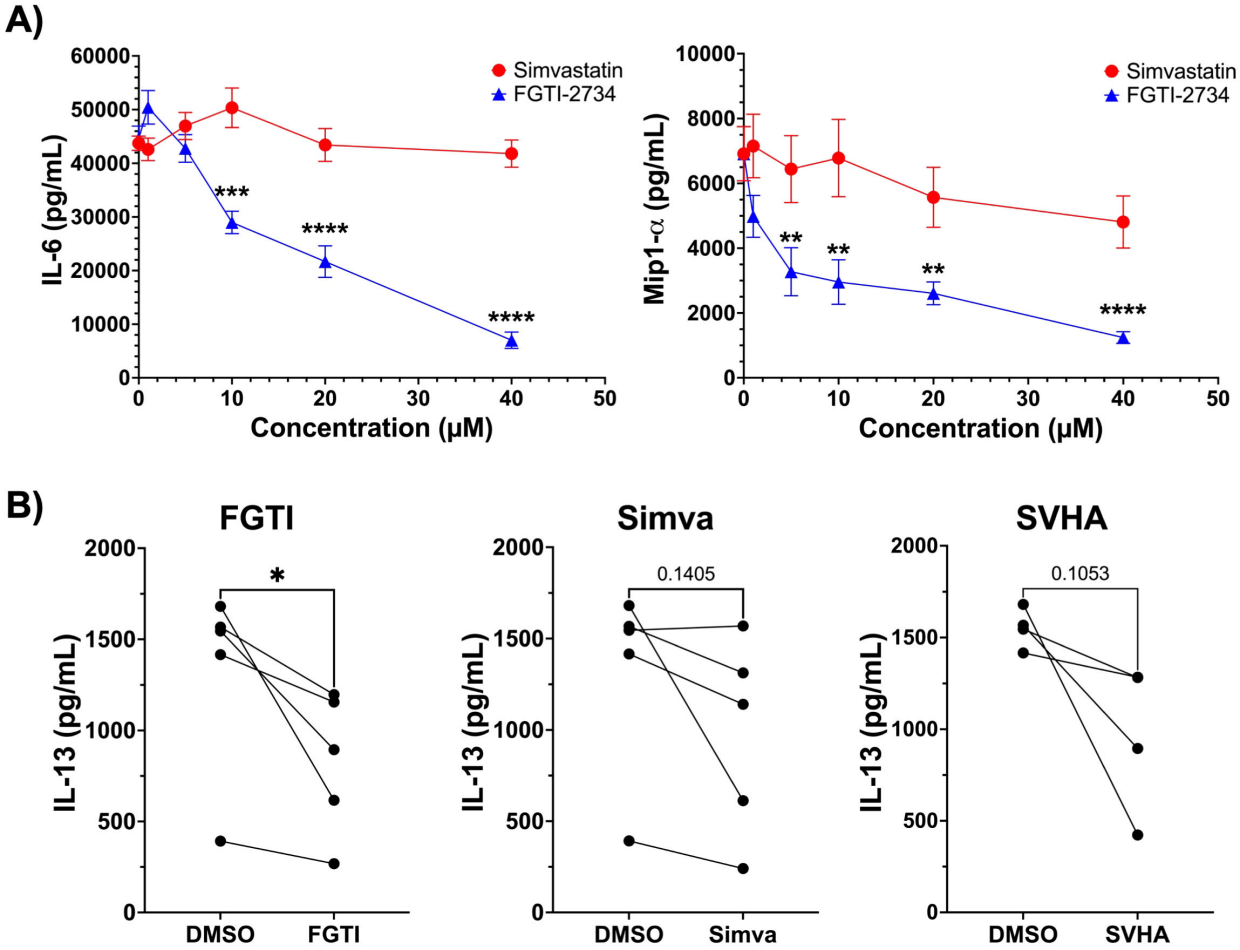
Genetic background affects the response to simvastatin but not FGTI-2734. **(A)** 129/Sv/J BMMC were treated with simvastatin or FGTI-2734 at 0, 1, 5, 10, 20, and 40μM for 24 hours, then stimulated with IL-33 (100ng/mL) for 16 hours. ELISA analysis was used to determine supernatant IL-6 and MIP-1a concentrations. Data are from 3 independent experiments using 3 independent BMMC populations analyzed in replicates by 1-way ANOVA with Dunnett’s post-hoc test and multiple comparisons correction. All comparisons are relative to the “0” control group. (B) HuMC were treated with FGTI-2734 (10μM), simvastatin (20μM), or SVHA (20μM) for 24 hours and then stimulated with IL-33 (100ng/ml) for 16 hours. Supernatant cytokines were measured by ELISA. Data represents 2 experiments. Each pair of icons is from an individual donor analyzed by paired t-Test between DMSO and FGTI-treated samples. *p< 0.05, ***p< 0.001, ****p< 0.0001, and no markers meaning non-significant.

We previously found that statin effects can vary with genetic background. For example, the 129/SvJ strain showed complete resistance to fluvastatin effects on IgE-induced mast cell function (25, 26). We assessed whether genetic background plays a role in the BMMC response to simvastatin and FGTI-2734. We found that simvastatin had no inhibitory effect on IL-33-induced IL-6 or MIP-1α (CCL3) release in BMMCs derived from 129/SvJ mice, while FGTI-2734 inhibition was intact (**Figure 4A**). We noted similar variability when studying human mast cells from 5 donors. While there was a general trend towards inhibition with simvastatin or simvastatin hydroxy acid treatment, only FGTI-2734 consistently reduced IL-33-mediated IL-13 secretion (**Figure 4B**). These data suggest that genetic background may alter statin effects more than FGTI-2734 effects.

### Isoprenylation inhibition does not inhibit IL-33 signaling through MAPKs or NFκB

**Figure 5:**
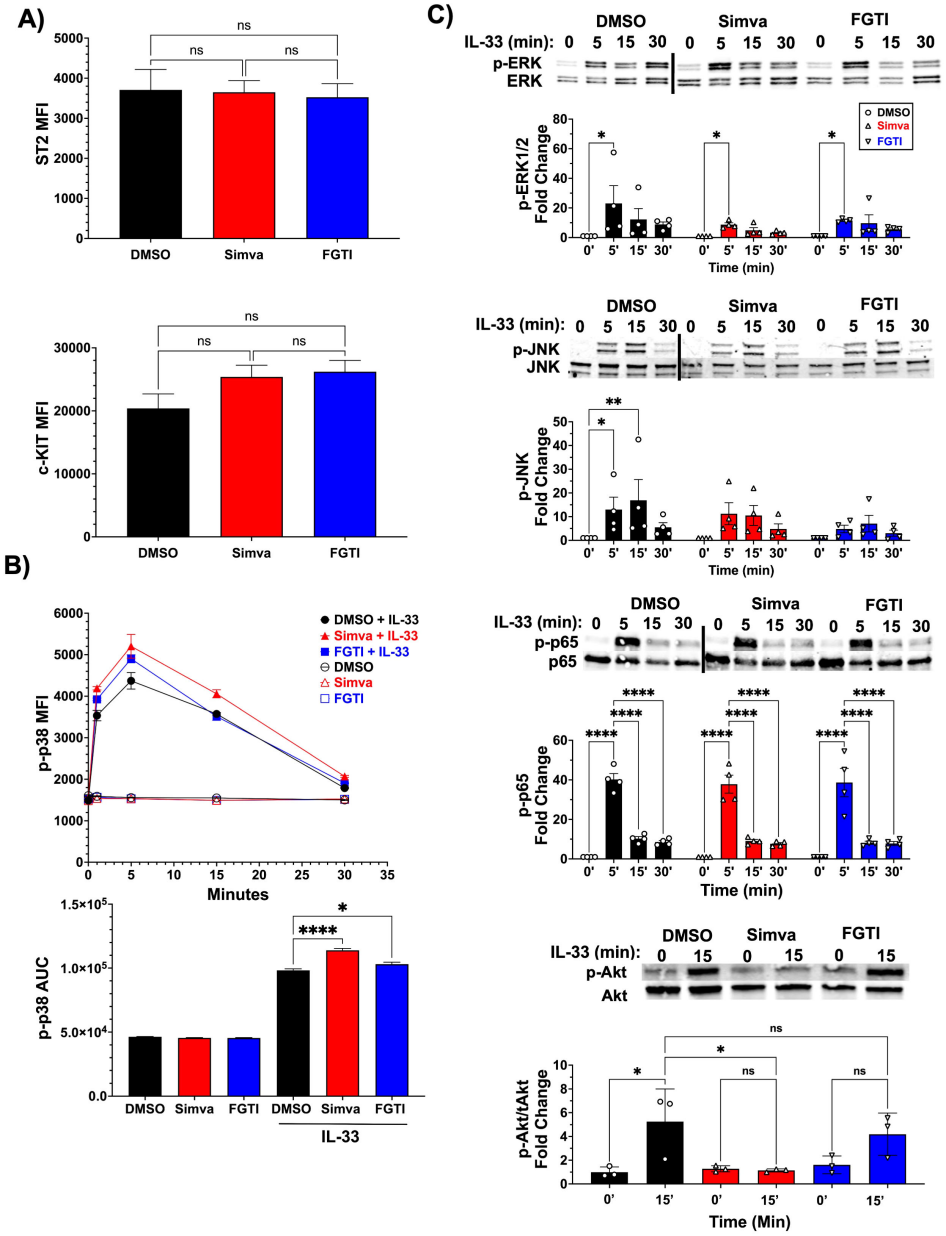
Simvastatin but not FGTI-2734 inhibits IL-33-mediated Akt activation. **(A)** C57BL/6J BMMC were treated with simvastatin (20μM), or FGTI-2734 (10μM) for 24 hours. Surface expression of ST2 and c-KIT were measured by flow cytometry. Data shown are from 4 experiments using at least 3 biological replicates each and compared by 1-way ANOVA with Dunnett’s post-hoc test and multiple comparisons correction. **(B)** C57BL/6J BMMC were treated with FGTI-2734 (10μM) or simvastatin (20μM) for 24 hours, then stimulated with IL-33 (100ng/mL). Phosphorylation of p38 was measured by flow cytometry. Data shown are from triplicate samples of 3 biological replicates. “AUC” is area under the curve analysis for the curves shown above. **(C)** C57BL/6J BMMC were treated with FGTI-2734 (10μM) or simvastatin (20μM) for 24 hours, then stimulated with IL-33 (100ng/mL). Phosphorylation of ERK1/2, JNK, p65, and Akt was measured by western blot. Data shown are from 3–5 experiments. Each point represents a BMMC population. Bars or icons throughout the figure represent the mean standard error of each sample set. *p< 0.05, **p< 0.01, ****p< 0.0001, and n.s. or absent markers meaning non-significant.

IL-33-ST2 signaling canonically activates the MyD88/IRAK/TRAF6 module, leading to signaling by MAP kinases and the transcription factors NFκB and AP-1 (21, 32). To determine if this signaling cascade is affected by isoprenylation inhibition, we first assessed the expression of the ST2 receptor on the surface of mast cells treated with simvastatin and FGTI-2734. BMMC were treated with drugs for 24 hours before staining for the ST2 receptor and expression levels were measured by flow cytometry. Results showed that ST2 surface expression was unchanged by simvastatin or FGTI-2734 (**Figure 5A**). We also measured c-KIT receptor expression, which binds stem cell factor, and has been shown to enhance IL-33-ST2 function signaling (33). c-KIT expression also showed no change in the presence of either drug (**Figure 5A**).

After assessing receptor expression, we moved down the cascade and measured phosphorylation of the MAPKs. Flow cytometry was used to assess phosphorylation of p38 MAPK. Gating strategy for this stain is shown in **Supplementary Figure 5**. BMMC were pretreated with drugs for 24 hours as before and activated with IL-33 at different time points, before being stained for phospho-p38. IL-33 induced strong p38 phosphorylation that peaked at 5 minutes and then steadily declined to baseline by 30 minutes (**Figure 5B**). Both the simvastatin and FGTI-2734 groups showed nearly identical phosphorylation events as the vehicle control group, indicating they did not suppress IL-33-induced p38 activation. Phosphorylation of the MAPKs ERK and JNK were measured by Western blot after similar treatment and activation with IL-33. Both kinases were activated by IL-33 and although a trend towards reduced phosphorylation was noted, neither kinase was significantly suppressed under these conditions (**Figure 5C**). Similarly, phosphorylation of NFκB, which branches off separately from TRAF6 and is not reliant on the MAPKs, was also unaffected by simvastatin or FGTI-2734 (**Figure 5C**). Finally, we found that IL-33-induced Akt phosphorylation was inhibited by simvastatin but not by FGTI-2734 (**Figure 5C**). These data suggest a possible mechanism by which simvastatin acts, but we have found no mechanism for FGTI-2734. These data also indicate that the canonical MAPK and NFκB pathways downstream of ST2 are not significantly inhibited by simvastatin or FGTI-2734, suggesting a novel means of suppression.

### Simvastatin and FGTI-2734 effects are consistent in eosinophils

**Figure 6:**
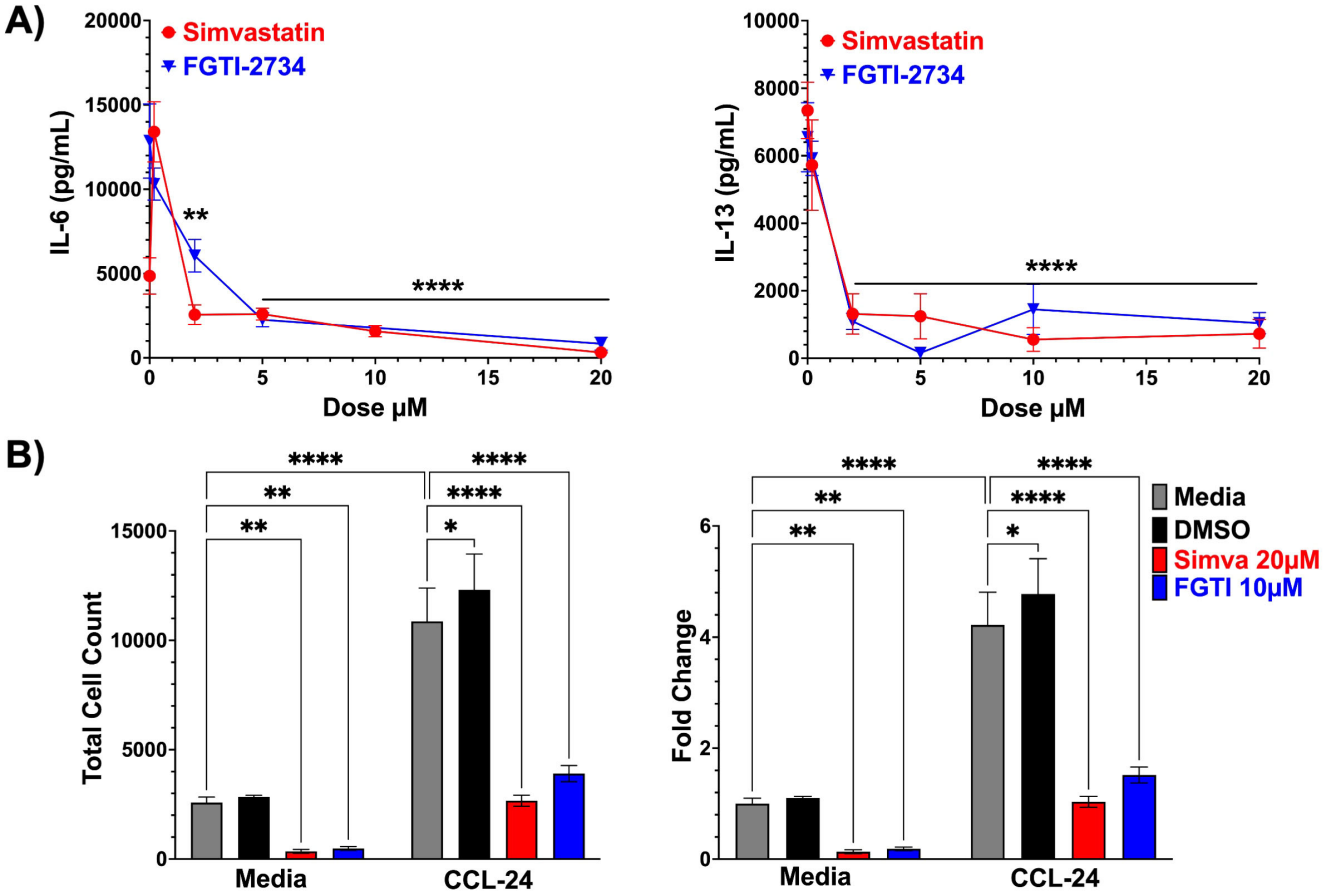
Simvastatin and FGTI-2734 inhibit eosinophil responses to IL-33 and chemokine-induced migration. **(A)** C57BL/6J BMDE were treated with FGTI-2734 or simvastatin at 0.2, 2, 5, 10, and 20μM for 24 hours, then stimulated with IL-33 (100ng/mL) for 16 hours. ELISA analysis was used to determine supernatant concentrations of IL-6, and IL-13. Data shown are from at least 3 biological replicates run in triplicate and analyzed by 1-way ANOVA relative to the matched “0” control groups, with Dunnett’s post-hoc test and test and multiple comparisons correction. **(B)** C57BL/6J BMDE were treated with simvastatin (20μM), or FGTI-2734 (10μM) for 24 hours. Transwell migration of BMDE to the lower chamber, stimulated by media or CCL24 (14.6nM), was measured by cell count by flow cytometry after 2.5 hours. Data are representative of 2 experiments using 3 biological replicates run in triplicate and analyzed by 1-way ANOVA with Dunnett’s post-hoc test and multiple comparisons correction. Bars or icons throughout the figure represent the mean standard error of each sample set. *p< 0.05, **p< 0.01, ****p<0.0001, and no markers meaning non-significant.

We hypothesized that IL-33-mediated activation of eosinophils, a key effector cell of allergic inflammation, could also be suppressed by simvastatin and FGTI-2734 (34). We cultured bone marrow-derived eosinophils (BMDE) and measured IL-33-induced cytokine production after treating with increasing drug concentrations. Example flow staining for BMDE cell surface markers is shown in **Supplementary Figure 6**. IL-6 and IL-13 secretion was completely suppressed by as little as 2µM of simvastatin or FGTI-2734 (**Figure 6A**).

One of the key characteristics of eosinophils that contributes to type 2 inflammation is their ability to migrate into inflamed tissue (23, 35). We first sought to test the effects of simvastatin and FGTI-2734 on IL-33-induced eosinophil migration. However, using a transwell assay, we were unable to elicit IL-33-induced migration (not shown). Therefore, we tested the response to CCL24, a chemokine known to induce eosinophil recruitment and whose production can be increased by IL-33 (36). We assessed BMDE migration to CCL24 and found that both total cell counts and fold migration increased toward CCL24, which was not affected by the DMSO vehicle (**Figure 6B**). In contrast, both simvastatin and FGTI-2734 reduced CCL24-mediated migration to nearly baseline (**Figure 6B**). We noted that these effects did not correlate with a significant decrease in eosinophil viability or expression of the CCL24 receptor, CCR3, suggesting that the drugs inhibited CCR3 signaling (**Supplementary Figure 7** and data not shown). Overall, these data support the hypothesis that isoprenylation plays a key role in eosinophil responses to IL-33 as well as chemokine-induced migration.

### FT and GGT dual inhibition but not simvastatin blocks IL-33 mediated function *in vivo*

To investigate the possible role isoprenylation plays in IL-33-mediated inflammation *in vivo*, we conducted a model of IL-33-induced peritonitis. IL-33 injected intraperitoneally once a day over six days has been shown to induce significant cellular recruitment into the peritoneal cavity, including neutrophils and eosinophils (37, 38).

**Figure 7:**
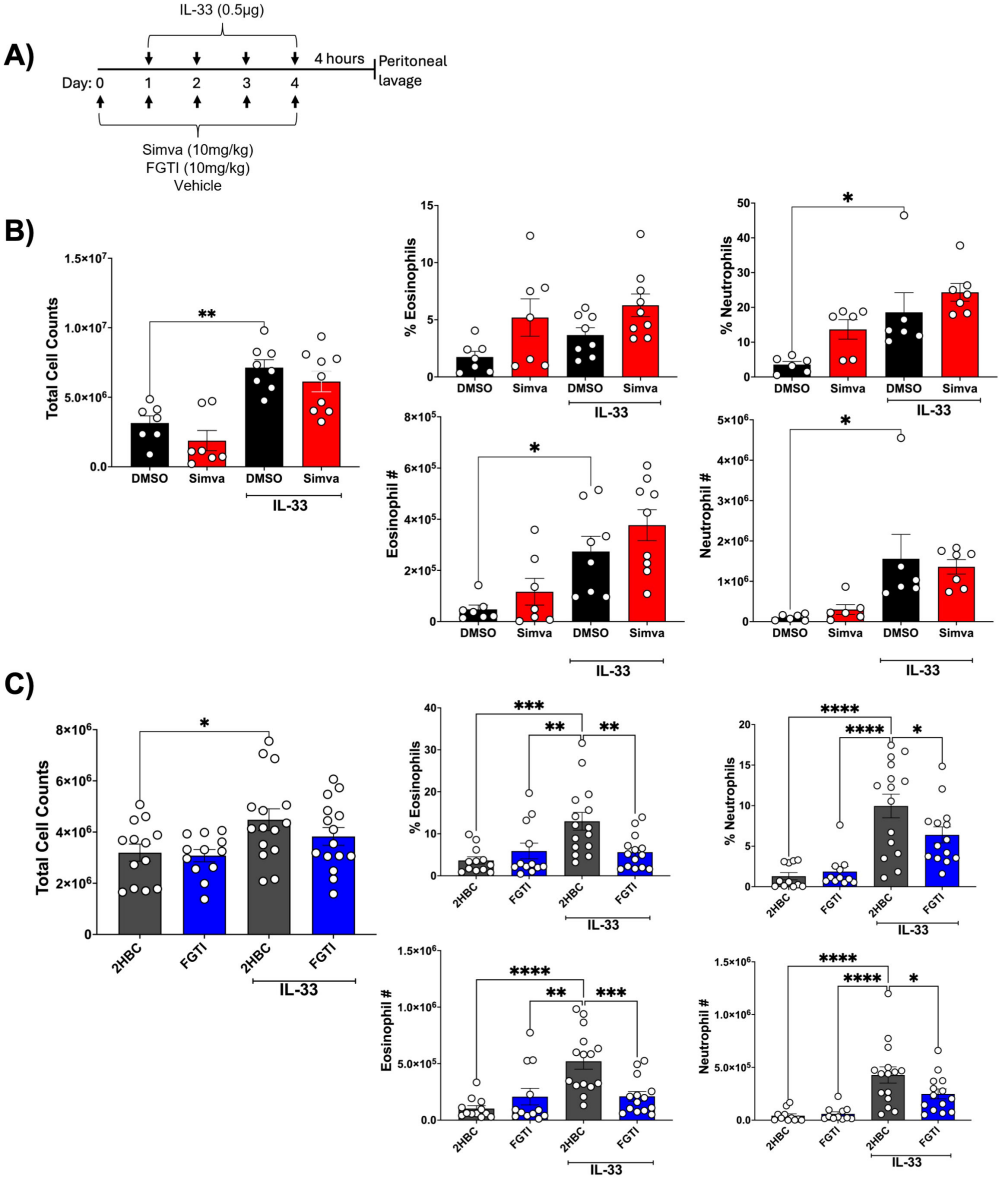
FGTI-2734 inhibits IL-33-mediated peritonitis *in vivo*. **(A)** Experimental schematic showing C57BL/6J mice were injected IP with vehicle, simvastatin (10mg/kg), or FGTI-2734 (10mg/kg) on days 0 – 4. IP injections of IL-33 (0.5µg) were given on days 1 – 4. Mice were euthanized 4 hours after final injections and peritoneal lavages were collected for analysis by flow cytometry. **(B, C)** Cell counts were obtained by automated cell counter. Cell percent and number was obtained by flow cytometry staining for eosinophils (CD11b^+^, SiglecF^+^) and neutrophils (CD11b^+^, Ly6G^+^). Data are representative of 3 experiments; each dot represents a single mouse. P values were obtained from 1-way ANOVA with Dunnett’s *post-hoc* test and multiple comparisons correction. Bars throughout the figure represent the mean standard error of each sample set. *p< 0.05, **p< 0.01, ***p< 0.001, ****p<0.0001, and no markers meaning non-significant.

To test simvastatin’s ability to suppress infiltration of immune cells into the peritoneal cavity, we intraperitoneally administered simvastatin (10 mg/kg) or DMSO (vehicle) on day zero, then for 4 more days, injected simvastatin +/- IL-33 (0.5 μg/day). Four hours after the final injection, peritoneal lavage was collected to analyze cellular infiltrate (**Figure 7A**). Total cellular content was modestly increased by the administration of IL-33, which was not significantly changed by simvastatin or FGTI-2734. Peritoneal cells were stained for eosinophils (CD45+, CD11b+, SiglecF+) and neutrophils (CD45+, CD11b+, Ly6G+). The percentage and number of cells were increased by IL-33 (**Figure 7B**). Simvastatin had no significant effect on eosinophil or neutrophil influx (**Figure 7B**). We noted that simvastatin unexpectedly tended to increase eosinophil and neutrophil percentage and number. Although this did not reach statistical significance, it suggests the drug has an unanticipated ability to recruit innate immune cells. In contrast, FGTI-2734 reduced both the percentage and number of eosinophils and neutrophils to nearly baseline levels (**Figure 7C**). Collectively, our data show that FGTI-2734 can suppress IL-33-mediated inflammatory responses *in vivo*, while under similar conditions, simvastatin had no effect. These results support the hypothesis that isoprenylation is a critical feature of IL-33-induced innate inflammation.

## Discussion

Type 2 inflammatory diseases are increasing in prevalence worldwide (39, 40). Current therapeutic interventions are effective for many patients, but partial symptom relief and treatment resistance are common (41). Therefore, it is important to investigate alternative options for the treatment of type 2 inflammation. Previously, our lab investigated the roles of statins and isoprenylation inhibitors in suppressing mast cell responses to the high affinity IgE receptor, FcϵRI (25, 26, 42). Statins are one of the most prescribed medications in the United States. In 2018-2019, 92M Americans used a statin, with simvastatin being the second-most common (34% of usage) (43). These drugs inhibit HMGCR, reducing cholesterol synthesis (8). We have shown that statins, particularly fluvastatin, can inhibit FcϵRI-mediated mast cell function (26). During this previous study, we determined that fluvastatin acts by suppressing isoprenylation, a pathway that branches off from cholesterol synthesis, rather than through reducing cholesterol content (26). Further investigation into isoprenylation revealed that both FT and GGT-I must be inhibited to optimally reduce FcϵRI-induced mast cell function (25). The previous studies focused on IgE signaling and exclusively addressed mast cells. We set out to determine if this pathway is also critical for IL-33 effects on mast cells and if the effects are consistent in other myeloid cells.

IL-33 is a potent driver of the Type 2 immune response, including eosinophil activation, infiltration, goblet cell hyperplasia, and cytokine production (44). An unusual cytokine that is constitutively found in the nucleus, IL-33 functions as an alarmin or DAMP that elicits type-2 inflammation when released from damaged cells (45). Our current work shows that simvastatin inhibits IL-33 mediated immune cell function by disrupting isoprenylation. This reflects similar findings we made on FcϵRI-mediated mast cell function, indicating the consistent importance of isoprenylation to mast cell function (25). In this study, we showed that simvastatin inhibited cellular function *in vitro*, but this effect was lost in an *in vivo* model of peritonitis. In contrast, FGTI-2734 inhibited immune cell infiltration in the same model, indicating that isoprenylation is important to IL-33-induced inflammation. Potential explanations for this discrepancy are the method of drug administration, differential drug metabolism, or that simvastatin is unexpectedly inflammatory in the peritoneum. In fact, we did note a trend toward greater eosinophil and neutrophil content when simvastatin was administered in the absence of IL-33. In support of this possibility, statins are well known to cause myositis in perhaps 15% of patients, and even autoimmune disease in rare cases (46, 47). Understanding how statins affect specific cell populations, including eosinophil and neutrophils, could be an important aspect of drug biology. Ultimately, our *in vivo* model supported the hypothesis that targeting isoprenylation can reduce IL-33-mediated inflammation but did not support simvastatin as a means of doing so. Additional models and routes of drug delivery should be examined to determine if simvastatin has potential for drug repurposing. Importantly, our data support exploration of how isoprenylation controls IL-33 responses, including the unknown protein targets that participate in ST2 signaling.

We found that the effects of isoprenylation inhibition are not mast cell-restricted. ST2 is expressed on many immune cells, including eosinophils (44). Eosinophil function was decreased, including not only IL-33-mediated cytokine production but also CCL24-induced migration. Eosinophils seemed to be very sensitive to isoprenylation blockade, with a nearly complete loss of IL-33-induced IL-6 and IL-13 secretion at drug concentrations under 5μM. This is encouraging, given that eosinophils are a critical component of allergic inflammation and the hallmark of many allergic diseases (34, 35, 48). Importantly, we noted no change in cell viability. This suggests that eosinophils are heavily reliant on isoprenylated proteins for their response to IL-33 as well as CCL24. Identifying these proteins could reveal novel aspects of eosinophil biology and perhaps offer new disease targets.

Our study had limitations. First, we used culture conditions that included SCF, since this cytokine acts on many mast cell populations *in vivo* and augments mast cell growth *in vitro*. It is worth noting that SCF increased IL-33-mediated TNF production without affecting IL-6 in a previous study (33). More importantly, we did not reveal a complete mechanism by which simvastatin and FGTI-2734 disrupt mast cell function. We linked simvastatin effects to isoprenylation inhibition and reduced IL-13 and TNFα transcription. Interestingly, IL-6 message was increased but nevertheless, cytokine secretion was diminished. We considered that if cytokine transcription and secretion were reduced, ST2-activated signaling proteins would likely be suppressed. The canonical IL-33 signaling pathway employs MyD88/IRAK/TRAF6 and the activation of MAPKs and NFκB that lead to transcription of inflammatory cytokines (32). Yet we found no difference in phosphorylation of MAPKs or the NFκB protein p65. We did note that simvastatin, but not FGTI-2734, inhibited IL-33-mediated Akt phosphorylation. We previously showed that under conditions of high SCF stimulation for 3 days, Akt impedes IL-33-mediated mast cell cytokine production (49). This outcome was surprising and is counter to the prevailing literature suggesting Akt promotes IL-33-ST2 function (50–54). Thus, it is possible that Akt has distinct effects depending on conditions including SCF-Kit signaling strength. It is interesting to note that although NFκB is a critical factor in IL-33-mediated cytokine production, we found no change in this pathway upon drug treatment. Similarly, Nakajima and coworkers noted that resveratrol decreased IL-33-induced mast cell cytokine production without inhibiting NFκB (51). This group also noted decreased Akt activation. Since FGTI-2734 had no effect on Akt, we conclude that inhibiting the Akt pathway is not a consistent outcome of targeting isoprenylation. A complete mechanism explaining our data will require further study.

Given the importance of prenylation to Ras family proteins, one could logically conclude that Ras protein inhibition explains at least some of the effects we have observed. Despite well-known links between Ras family proteins and MAPK signaling, there is surprisingly little published evidence that IL-33-ST2 signaling employs the Ras pathway. Suzuki et al. showed that the protein Spred1, which can inhibit Ras function by recruiting a Ras GTPase activating protein, limits IL-33 effects on innate lymphoid cells. Others have shown that Ras-mediated transformation induces IL-33 expression (55–57). No specific Ras family protein has yet been shown critical for IL-33-induced function, despite the likelihood that this is the case. Our future work is partly focused on mapping Ras protein activation by IL-33.

Like our studies with FcϵRI, we found that statin effectiveness was tied to genetic background (25, 26). The 129/SvJ strain showed complete resistance to fluvastatin in our previous studies and to simvastatin in the current work. More importantly, primary human mast cells also showed a varied simvastatin response that did not reach statistical significance. This is important and perhaps anticipated, as statin resistance among patients using the drugs to reduce cholesterol is also common (58). We previously found that fluvastatin treatment induced a 3-fold increase in HMGCR expression among 129/SvJ but not C57BL/6 BMMC, suggesting a mechanism of drug resistance (26). This variability may explain the difficulties in demonstrating a consistent effect of statins in allergic disease (59, 60). It is important to note that FGTI-2734 effects in our previous and current studies have been consistent across mouse strains and human donors. We also noted that simvastatin and FGTI-274 were effective on mast cells and eosinophils cultured from male or female mice; no sex-restricted effect was found (not shown). It is therefore possible that targeting isoprenylation could be a reliable means of suppressing allergic inflammation.

In summary, this study adds to the growing evidence that isoprenylation is an essential pathway for immune cell function, by demonstrating that IL-33-mediated inflammation is suppressed by isoprenylation inhibition. While there is still a need to determine a mechanism and fully characterize effects *in vivo*, these data have implications for drug repurposing and development. Moreover, our findings support further work to identify isoprenylated proteins playing critical roles in allergic inflammation.

## Data Availability

The raw data supporting the conclusions of this article will be made available by the authors, without undue reservation.
