# Supplementary material for: IL-33-mediated mast cell and eosinophil function requires isoprenylation
Source: Front Immunol. 2025 Dec 11;16:1662170. doi: 10.3389/fimmu.2025.1662170 (PMC12738314; doi:10.3389/fimmu.2025.1662170)
Supplement: Supplementary file 1 [file Presentation1.pptx]

## Slide 1
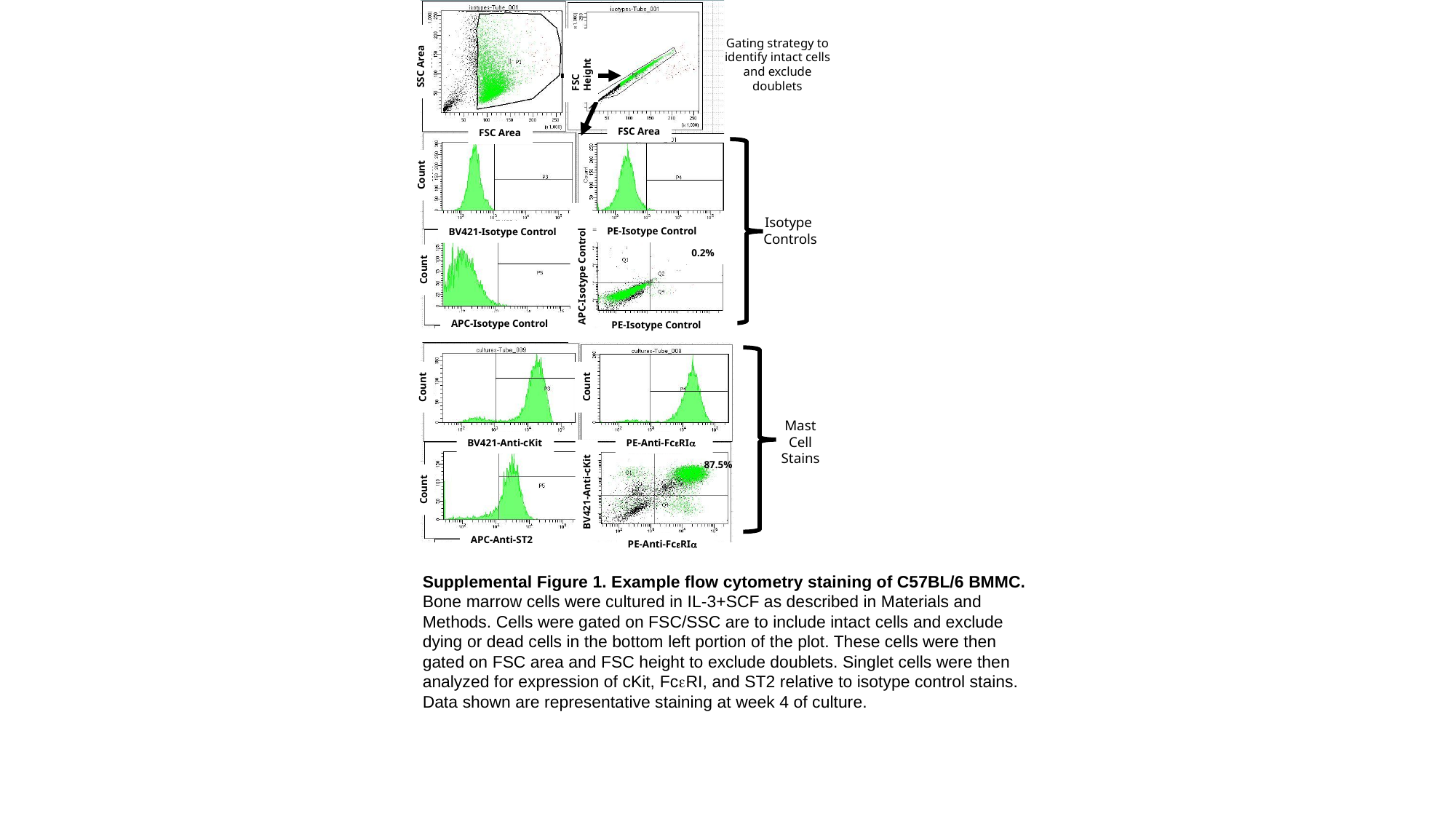

Gating strategy to identify intact cells and exclude doublets
SSC Area
FSC Height
FSC Area
FSC Area
Count
Isotype
Controls
PE-Isotype Control
BV421-Isotype Control
0.2%
APC-Isotype Control
Count
APC-Isotype Control
PE-Isotype Control
Count
Count
Mast Cell Stains
BV421-Anti-cKit
PE-Anti-FceRIa
87.5%
Count
BV421-Anti-cKit
APC-Anti-ST2
PE-Anti-FceRIa
Supplemental Figure 1. Example flow cytometry staining of C57BL/6 BMMC. Bone marrow cells were cultured in IL-3+SCF as described in Materials and Methods. Cells were gated on FSC/SSC are to include intact cells and exclude dying or dead cells in the bottom left portion of the plot. These cells were then gated on FSC area and FSC height to exclude doublets. Singlet cells were then analyzed for expression of cKit, FceRI, and ST2 relative to isotype control stains. Data shown are representative staining at week 4 of culture.

## Slide 2
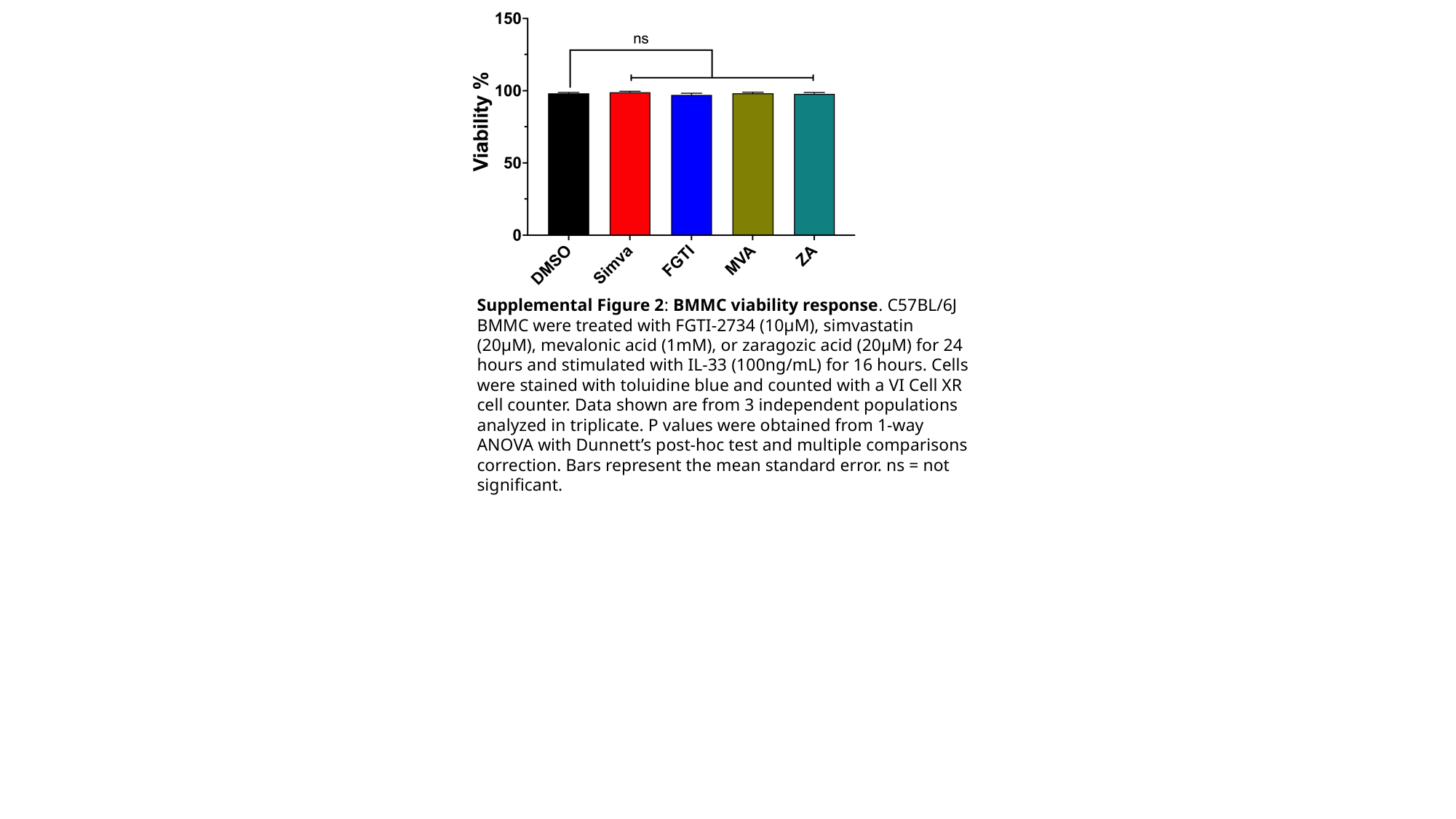

Supplemental Figure 2: BMMC viability response. C57BL/6J BMMC were treated with FGTI-2734 (10µM), simvastatin (20µM), mevalonic acid (1mM), or zaragozic acid (20µM) for 24 hours and stimulated with IL-33 (100ng/mL) for 16 hours. Cells were stained with toluidine blue and counted with a VI Cell XR cell counter. Data shown are from 3 independent populations analyzed in triplicate. P values were obtained from 1-way ANOVA with Dunnett’s post-hoc test and multiple comparisons correction. Bars represent the mean standard error. ns = not significant.

## Slide 3
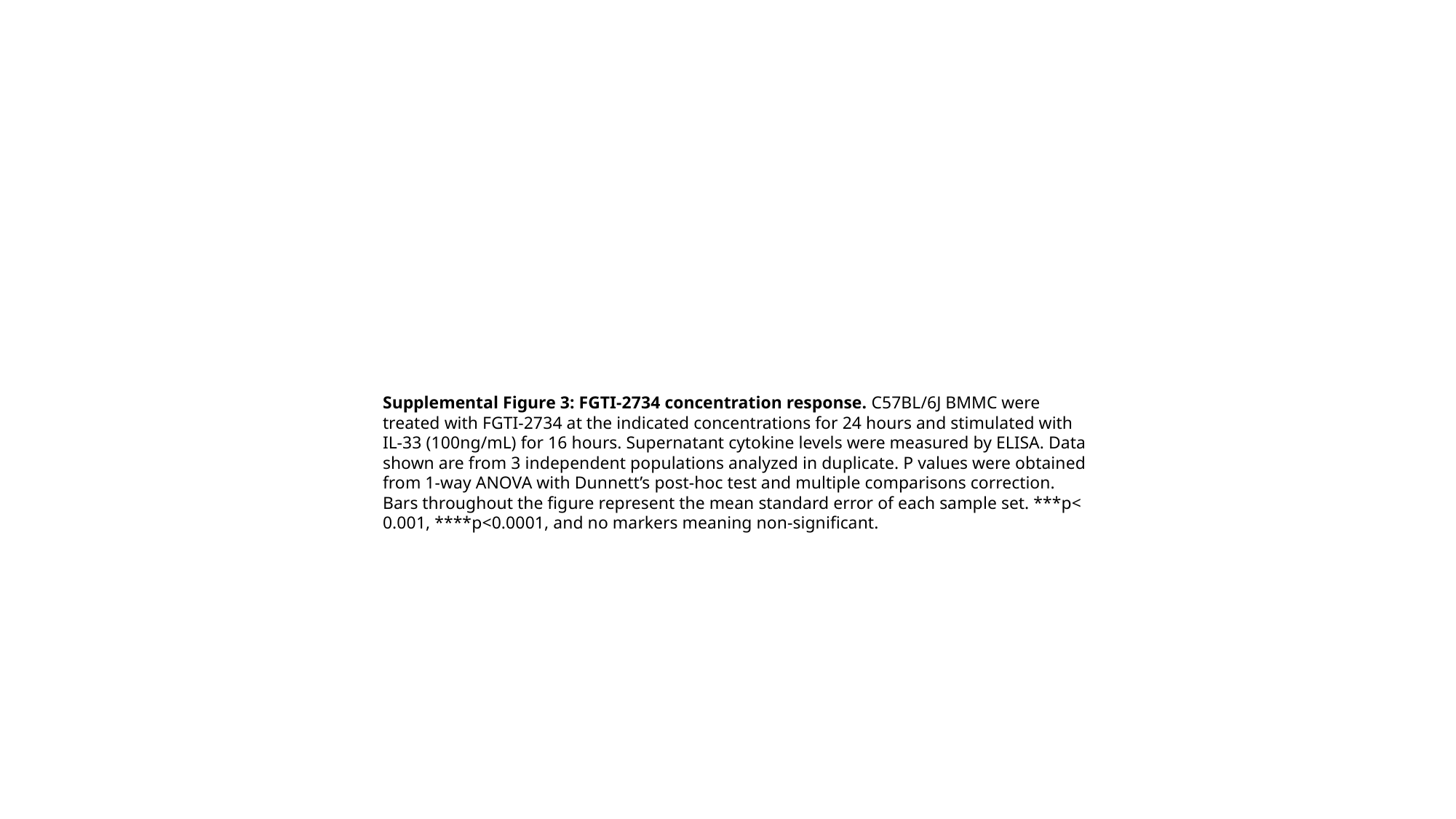

Supplemental Figure 3: FGTI-2734 concentration response. C57BL/6J BMMC were treated with FGTI-2734 at the indicated concentrations for 24 hours and stimulated with IL-33 (100ng/mL) for 16 hours. Supernatant cytokine levels were measured by ELISA. Data shown are from 3 independent populations analyzed in duplicate. P values were obtained from 1-way ANOVA with Dunnett’s post-hoc test and multiple comparisons correction. Bars throughout the figure represent the mean standard error of each sample set. ***p< 0.001, ****p<0.0001, and no markers meaning non-significant.

## Slide 4
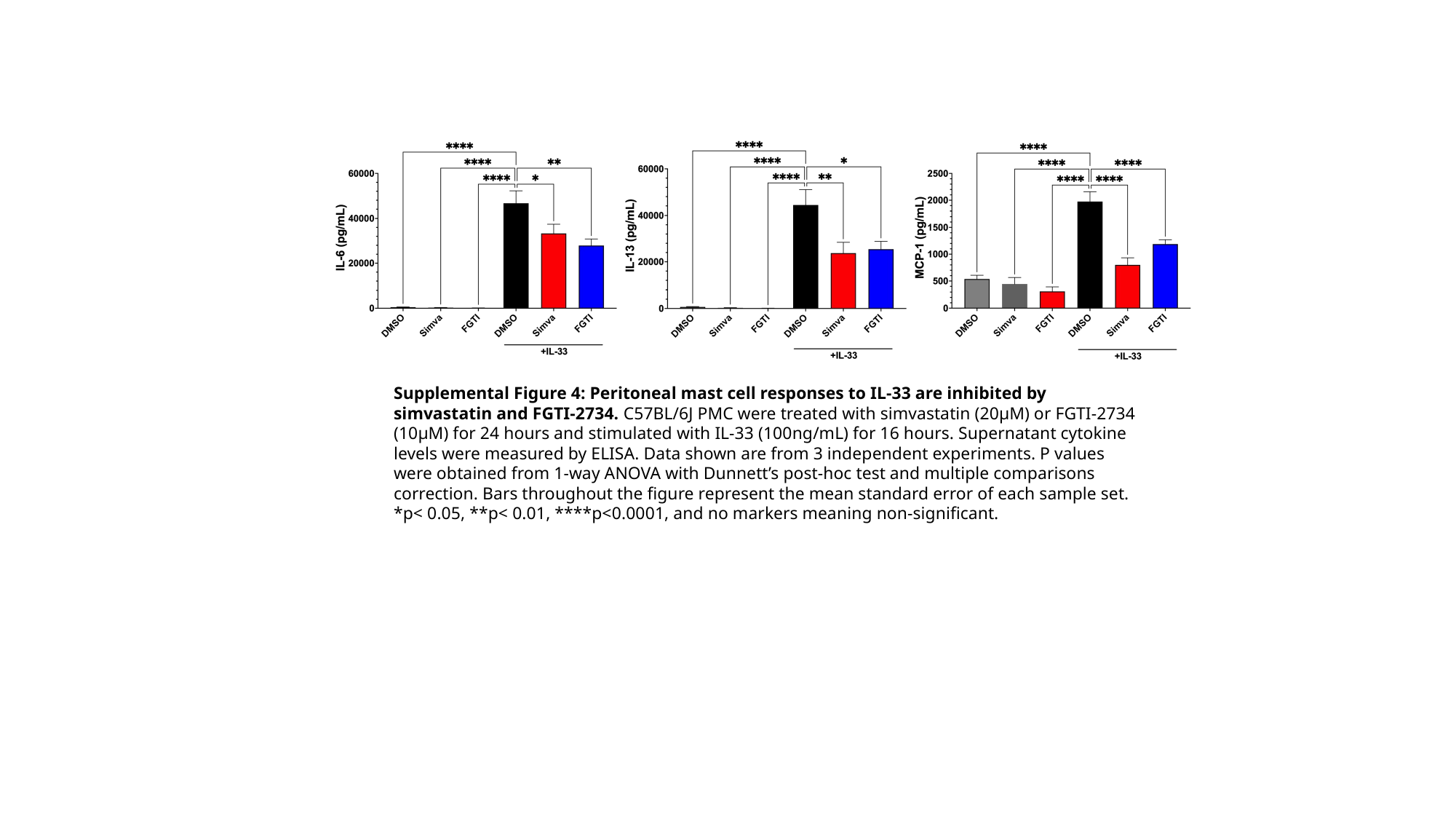

Supplemental Figure 4: Peritoneal mast cell responses to IL-33 are inhibited by simvastatin and FGTI-2734. C57BL/6J PMC were treated with simvastatin (20µM) or FGTI-2734 (10µM) for 24 hours and stimulated with IL-33 (100ng/mL) for 16 hours. Supernatant cytokine levels were measured by ELISA. Data shown are from 3 independent experiments. P values were obtained from 1-way ANOVA with Dunnett’s post-hoc test and multiple comparisons correction. Bars throughout the figure represent the mean standard error of each sample set. *p< 0.05, **p< 0.01, ****p<0.0001, and no markers meaning non-significant.

## Slide 5
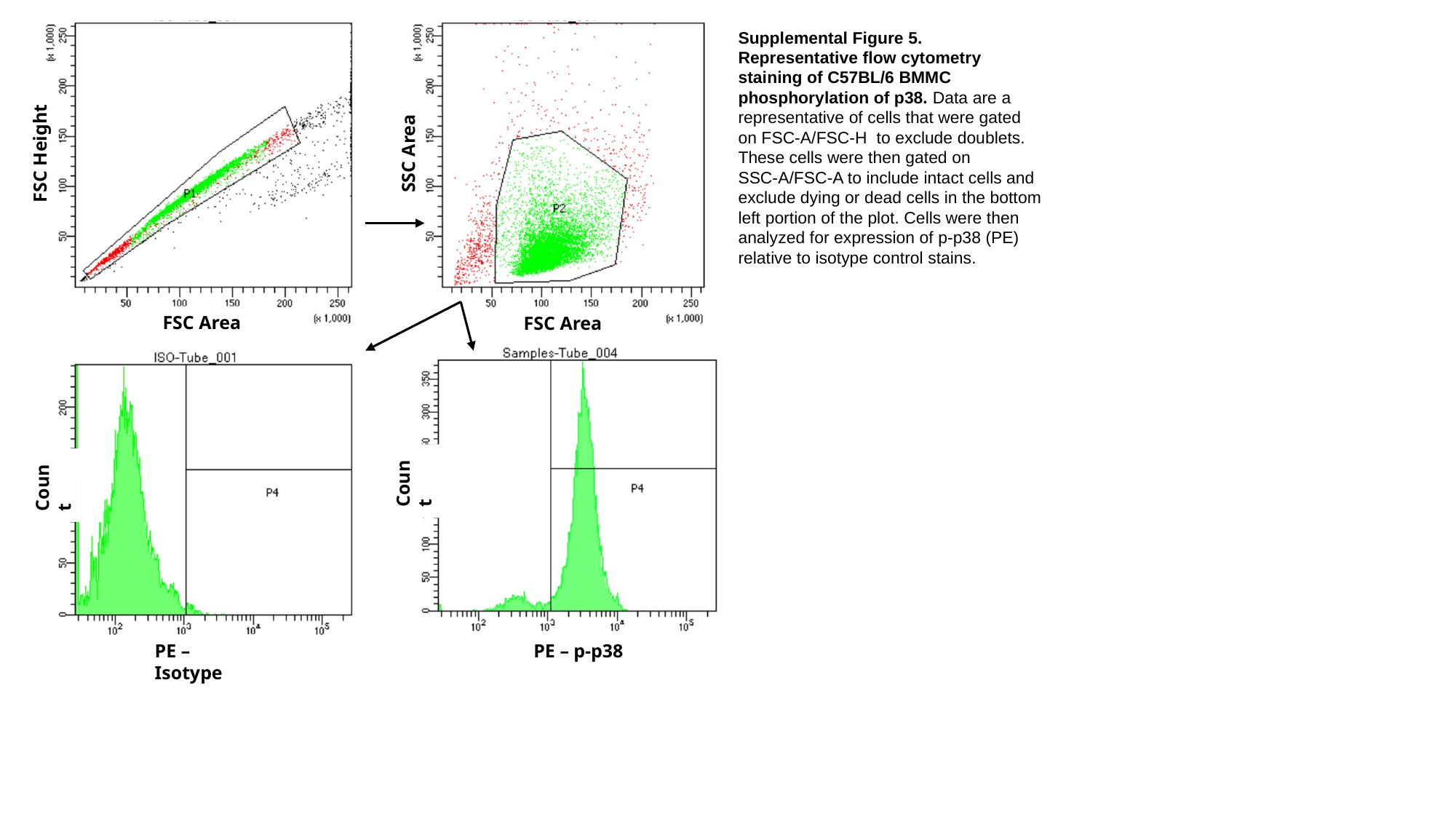

Supplemental Figure 5. Representative flow cytometry staining of C57BL/6 BMMC phosphorylation of p38. Data are a representative of cells that were gated on FSC-A/FSC-H to exclude doublets. These cells were then gated on SSC-A/FSC-A to include intact cells and exclude dying or dead cells in the bottom left portion of the plot. Cells were then analyzed for expression of p-p38 (PE) relative to isotype control stains.
FSC Height
SSC Area
FSC Area
FSC Area
Count
Count
PE – p-p38
PE – Isotype

## Slide 6
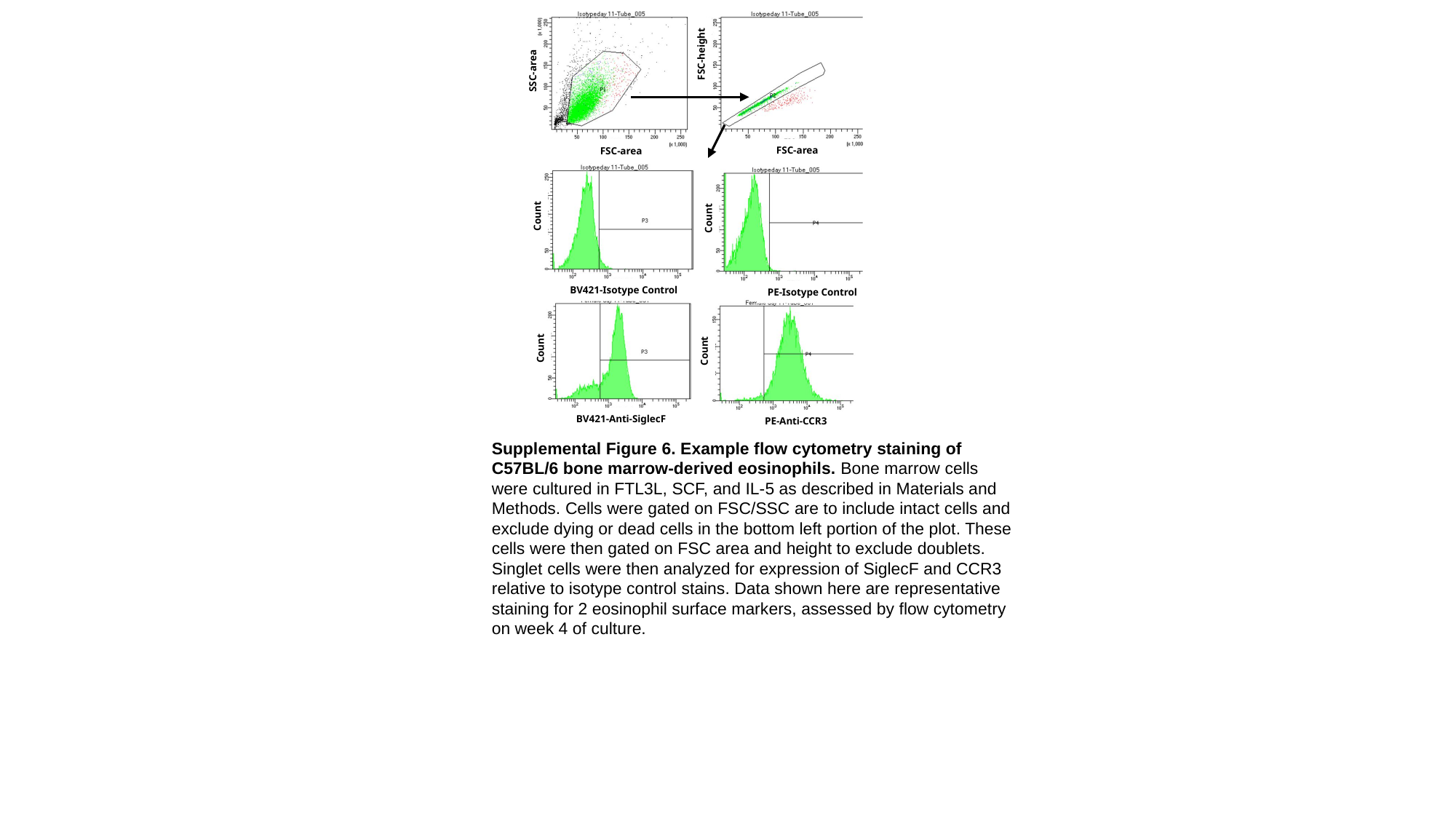

FSC-height
SSC-area
FSC-area
FSC-area
Count
Count
BV421-Isotype Control
PE-Isotype Control
Count
Count
BV421-Anti-SiglecF
PE-Anti-CCR3
Supplemental Figure 6. Example flow cytometry staining of C57BL/6 bone marrow-derived eosinophils. Bone marrow cells were cultured in FTL3L, SCF, and IL-5 as described in Materials and Methods. Cells were gated on FSC/SSC are to include intact cells and exclude dying or dead cells in the bottom left portion of the plot. These cells were then gated on FSC area and height to exclude doublets. Singlet cells were then analyzed for expression of SiglecF and CCR3 relative to isotype control stains. Data shown here are representative staining for 2 eosinophil surface markers, assessed by flow cytometry on week 4 of culture.

## Slide 7
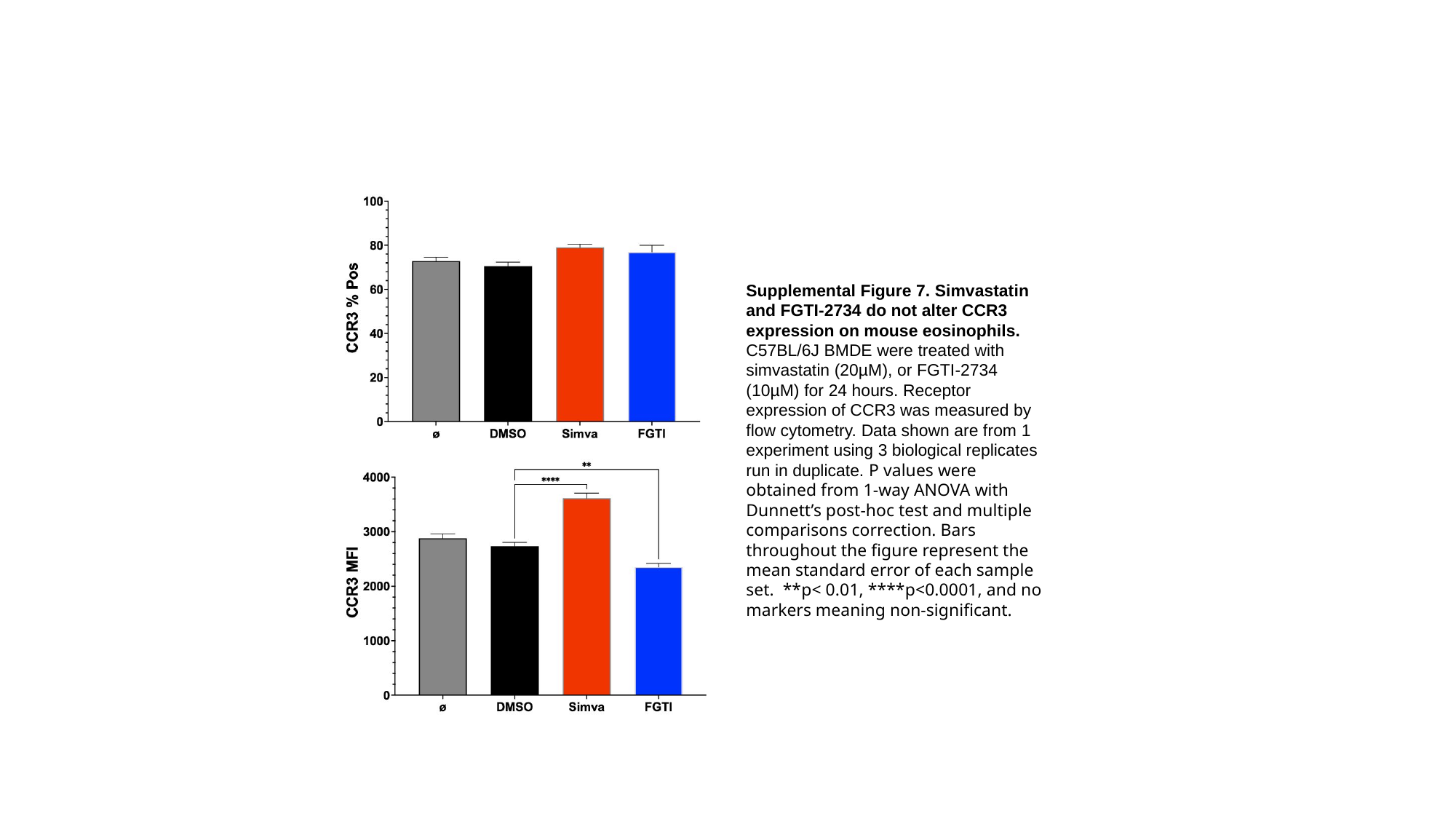

Supplemental Figure 7. Simvastatin and FGTI-2734 do not alter CCR3 expression on mouse eosinophils.
C57BL/6J BMDE were treated with simvastatin (20µM), or FGTI-2734 (10µM) for 24 hours. Receptor expression of CCR3 was measured by flow cytometry. Data shown are from 1 experiment using 3 biological replicates run in duplicate. P values were obtained from 1-way ANOVA with Dunnett’s post-hoc test and multiple comparisons correction. Bars throughout the figure represent the mean standard error of each sample set. **p< 0.01, ****p<0.0001, and no markers meaning non-significant.
